# Evidence for a “Pathogenic Triumvirate” in Congenital Hepatic Fibrosis in Autosomal Recessive Polycystic Kidney Disease

**DOI:** 10.1155/2016/4918798

**Published:** 2016-11-07

**Authors:** Lu Jiang, Pingping Fang, James L. Weemhoff, Udayan Apte, Michele T. Pritchard

**Affiliations:** Department of Pharmacology, Toxicology and Therapeutics, University of Kansas Medical Center, 3901 Rainbow Blvd, Kansas City, KS 66160, USA

## Abstract

Autosomal recessive polycystic kidney disease (ARPKD) is a severe monogenic disorder that occurs due to mutations in the* PKHD1* gene. Congenital hepatic fibrosis (CHF) associated with ARPKD is characterized by the presence of hepatic cysts derived from dilated bile ducts and a robust, pericystic fibrosis. Cyst growth, due to cyst wall epithelial cell hyperproliferation and fluid secretion, is thought to be the driving force behind disease progression. Liver fibrosis is a wound healing response in which collagen accumulates in the liver due to an imbalance between extracellular matrix synthesis and degradation. Whereas both hyperproliferation and pericystic fibrosis are hallmarks of CHF/ARPKD, whether or not these two processes influence one another remains unclear. Additionally, recent studies demonstrate that inflammation is a common feature of CHF/ARPKD. Therefore, we propose a “pathogenic triumvirate” consisting of hyperproliferation of cyst wall growth, pericystic fibrosis, and inflammation which drives CHF/ARPKD progression. This review will summarize what is known regarding the mechanisms of cyst growth, fibrosis, and inflammation in CHF/ARPKD. Further, we will discuss the potential advantage of identifying a core pathogenic feature in CHF/ARPKD to aid in the development of novel therapeutic approaches. If a core pathogenic feature does not exist, then developing multimodality therapeutic approaches to target each member of the “pathogenic triumvirate” individually may be a better strategy to manage this debilitating disease.

## 1. Introduction

Autosomal recessive polycystic kidney disease (ARPKD) is a rare genetic disorder that occurs in 1 : 20,000 live births. It develops* in utero* and is mainly diagnosed in pregnancy or in the immediate neonatal period. Among all affected patients, approximately 30% die shortly after birth, primarily of pulmonary insufficiency [1]. Patients who survive the neonatal period present with a broad spectrum of symptoms involving the kidneys, liver, and pancreas. Renal manifestations are characterized by the presence of cysts that are derived from dilated collecting ducts and distal tubules [2, 3]. A significant portion of patients will progress to end stage renal disease either during the first decade or during adolescence [4]. A minority of patients develop pancreatic abnormalities consisting of cysts and fibrosis [5, 6]. All patients with ARPKD develop some degree of congenital hepatic fibrosis (CHF), which, as the name would suggest, is present at birth. CHF is characterized by bile duct dilation resulting in eventual development of cysts and pericystic fibrosis in the liver [7, 8]. Accompanying cyst growth and fibrosis, recent reports suggest that inflammation is also present and likely contributes to disease pathogenesis and/or progression [9–12]. Aside from management of symptoms and liver and/or kidney transplant, no effective pharmacologic therapies exist for CHF/ARPKD [13].

Although CHF is most commonly thought to be associated with ARPKD, there are several cases reported in autosomal dominant polycystic kidney disease (ADPKD). The patients with ADPKD showed hepatic cysts and fibrosis at birth, which is consistent with symptoms described in CHF/ARPKD [14]. Other ciliopathies in which CHF is found include Meckel-Gruber syndrome [15], renal-hepatic-pancreatic dysplasia (an autosomal recessive disorder with renal dysplasia and pancreatic fibrosis) [16], and COACH syndrome (a subset of Joubert Syndrome Related Disorders, an autosomal recessive multisystemic disorder with cerebellar vermis hyperplasia, ataxia, and mental retardation) [17].

## 2. Gene Defects in ARPKD

CHF/ARPKD is caused by mutations in the* PKHD1* gene.* PKHD1* extends over 470 kb, includes a minimum of 86 exons, and encodes a 4,074-amino-acid protein called fibrocystin/polyductin. Fibrocystin is predicted to be a receptor-like protein that consists of a large glycosylated extracellular region, a single transmembrane domain, and a short cytoplasmic tail [18, 19]. Fibrocystin is expressed in the primary cilia of epithelial cells. Immunohistochemistry studies suggest that fibrocystin is located in renal collecting ducts and loops of Henle, pancreatic epithelial ducts, and hepatic biliary ducts [20]. Different mutations in* PKHD1* have been described in human ARPKD patients, including missense mutations, deletion/insertion mutations, and splicing mutations. Among all types of mutations found in* PKHD1*, about 45% of them are predicted to truncate fibrocystin [21]. Disease in patients carrying two truncating mutations is usually more severe, whereas patients bearing missense mutations exhibit a milder phenotype [22].

## 3. Current Therapies for ARPKD

There is currently no pharmacologic cure for CHF/ARPKD. Treatment mainly focuses on management of symptoms and includes therapies for cardiac hypertension, chronic liver/kidney disease, cholangitis, and portal hypertension [23]. Hypertension associated with chronic kidney disease occurs at the early stage of disease and is regulated by the renin-angiotensin system (RAS) [24]. Hypertension in ARPKD is treated empirically. Angiotensin converting enzyme (ACE) inhibitors and angiotensin II receptor blockers (ARBs) are considered the main treatment options in ARPKD patients [8, 25]. If kidney failure occurs, patients undergo dialysis or kidney transplantation. CHF/ARPKD can be accompanied by recurrent cholangitis and cholangiocarcinoma. Although the occurrence of recurrent cholangitis and cholangiocarcinoma is relatively rare, liver transplantation is indicated to decrease mortality [26, 27]. Other therapeutic strategies include targeting components of the cAMP signaling pathway since cAMP levels are increased in cyst wall epithelial cells (CWECs) and drive CWEC proliferation. Octreotide and pasireotide, two somatostatin analogs, decrease proliferation of PCK rat CWEC* in vitro* and inhibit hepatorenal cyst growth in PCK rats* in vivo* by reducing cAMP levels. Consistently, clinical trials in patients with polycystic liver disease (PLD) and ADPKD showed that octreotide or lanreotide is well tolerated and decreases total liver volume by 4%–6% [13].

## 4. Animal Models of ARPKD

A number of rodent models of human ARPKD exist to study the mechanisms of disease and to test therapeutic strategies (Table 1). One of the best-characterized models is the polycystic kidney (PCK) rat, derived from Sprague-Dawley (SD) rats at Charles River, Inc. [28]. The PCK rat carries a spontaneous splicing mutation, IVS35-2A→T, in the rat* Pkhd1* gene [19]. PCK rats bear hepatic and renal cysts and associated fibrosis, similar to human ARPKD [29]. The lifespan of a PCK rats is about 1.5 years, and they develop numerous cysts in kidneys and liver by one year of age [30]. In mice, the congenital polycystic kidney (*cpk*) mouse mimics human ARPKD. This mouse harbors a spontaneous mutation in* cpk* gene, the gene that encodes a 145-amino-acid protein termed cystin. Cystin is mainly located in the axoneme of the primary cilia found in the kidney proximal tubules and collecting ducts and in the cholangiocytes found in the liver [31, 32].* cpk* mice, on the BALB/c background, exhibit both renal and extrarenal manifestations associated with cystin mutations [33]. When on a C57Bl/6J background,* cpk* mice do not have extrarenal pathology [34], limiting the utility of this model for those interested in studying CHF/ARPKD. In addition, *Pkhd*1^del2/del2^ mouse model, which lacks exon 2 of the mouse* Pkhd1* gene, also reproduces the human ARPKD pathology. Female mice develop dilation of the renal proximal tubule and cysts by 3 months of age, whereas male mice are protected from renal cysts. Both genders develop hepatic cysts and fibrosis by 3 months as a result of biliary ductal plate malformation [35]. Another widely accepted murine model with* Pkhd1* mutation was generated by Christopher Ward and colleagues. In this model, the* Pkhd1* gene was transcriptionally silenced by inserting a loxP flanked STOP (LSL) cassette into intron-2. *Pkhd*1^LSL(−)/LSL(−)^ mice, both male and female, develop liver cysts and fibrosis at 3 months of age [36]. In addition, by disrupting exon 40, homozygous Pkhd1 mutant mice exhibit severe hepatic cysts and pericystic fibrosis in neonates due to biliary malformation in the embryo. However, the morphology and function in kidneys are not affected [37]. Another well-described murine model is the homozygous *Pkhd*1^*lacZ*/*lacZ*^ mice. They are widely accepted as an ARPKD mouse model due to the presence of both hepatic and renal manifestations [5]. *Pkhd*1^*del*4/*del*4^ mouse is described predominantly as a model for CHF, since the kidneys are unaffected by the mutation [38]. Among all rodent models for ARPKD, the PCK rat is one of the few commercially available models to date, and the phenotypic resemblance to human ARPKD makes it an incredibly valuable resource for CHF/ARPKD researchers.

## 5. Mechanisms of Cystogenesis in ARPKD

Although the mechanisms of cystogenesis are not well characterized in human ARPKD, a study using PCK rats suggested a possible link between cystogenesis and ciliary dysfunction [39]. Primary cilia, microtubule-based organelles, extend from the surface of eukaryotic cells. Primary cilia are nonmotile cilia containing a “9+0” axoneme, and function as mechano-, osmo-, and chemosensors that deliver signals from the extracellular environment into the cell [40]. The abnormal primary cilia in PCK rat cholangiocytes may compromise their sensory organelle function in response to fluid secretion or fluid flow. Recent research suggests that primary cilia are also important components of multiple signaling pathways such as the hedgehog and PDGF-A signaling pathways [41, 42].

Hepatic cyst development in human ARPKD patient is characterized by abnormal remodeling of ductal plate from the double cell layer. Clinical manifestations include dilated bile ducts, an increased number of bile ducts, and abnormal branching [8]. Whether the hepatic cysts are disconnected from the biliary as they grow remains to be studied although it is the case in ADPKD patients [43]. Cyst development in PCK rat liver has been well-described by Dr. La Russo's group [13, 44, 45]. They found that (1) hepatic cysts are derived from bile duct segments due to ductal plate malformation during development and (2) most cysts become isolated from biliary tree by 6 months of age [39]. Along with cyst formation, the mechanisms of cyst expansion are proposed to be the result of the following: (1) cholangiocyte hyperproliferation, (2) cell-matrix interactions, and (3) fluid secretion [46]. Many factors can regulate these processes through different signaling pathways and are briefly described below.

(1) Intracellular cyclic adenosine monophosphate (cAMP) is likely the major driver of hepatic cyst growth [13]. In addition, cAMP levels are elevated in PCK rat cholangiocytes as compared to cholangiocytes from control, SD rats. Octreotide, a somatostatin analog, reduces hepatic and renal cyst expansion in PCK rats by decreasing cAMP levels [47]. Another factor that contributes to CWEC proliferation is low intracellular [Ca^2+^], which is reduced in CWECs from PCK rat livers [48]. Activation of Trpv4, a calcium-permeable cation channel expressed in normal cholangiocytes, increases intracellular calcium levels and suppresses proliferation of cholangiocytes isolated from PCK rats* in vitro* [46]. Although the mechanisms of cyst growth have been well-described, whether targeting cAMP or intracellular [Ca^2+^] will prevent disease progression in humans is inconsistent [13]. (2) Remodeling of extracellular matrix includes alteration of extracellular matrix composition, basement membrane thickness, and the activities of matrix metalloproteases (MMPs) and their inhibitors, all of which can lead to cyst expansion [49]. (3) In ARPKD, little is known about how fluid secretion impacts hepatic cyst expansion. Previous data suggest that cystic epithelia can respond to secretin and secrete fluid through activating cAMP-dependent signaling pathway [50, 51].

In contrast to the origin of cyst development in liver, renal cysts in ARPKD are commonly described as dilated collecting ducts [52]. In contrast to what is observed in ADPKD, dilated collecting ducts and distal tubules lined with cuboidal or columnar epithelia remain connected to the urinary system [53]. It remains unclear in ARPKD whether or not cysts with squamous epithelia cells detach from the tubular segment from which they are derived [54]. Cyst formation and expansion are associated with increased proliferation of renal epithelial cells [55] and altered fluid secretion [56]. Recent data suggest that cAMP induces renal epithelial cell proliferation and promotes cyst growth by activating PKA/B-Raf/MAPK pathways in CWECs from ARPKD patients [57]. Similarly, renal epithelial cells also exhibit a lower level of intracellular [Ca^2+^] and sustained reduction of intracellular [Ca^2+^] in normal cells induces a cAMP-growth stimulated phenotype [57]. In addition, an increased level of epidermal growth factor (EGF) receptor is demonstrated in renal cyst fluid, which is consistent with an overexpression of EGF receptor (EGFR) mRNA and protein in renal epithelia in cpk mice [58]. The administration of EGFR tyrosine kinase inhibitor does not protect PCK rats from developing renal cysts, possibly due to an increased level of cAMP after treatment [59].

## 6. Mechanisms of Fibrosis in ARPKD

Liver fibrosis results from chronic liver injury in conjunction with the accumulation of extracellular matrix (ECM) proteins synthesized by myofibroblasts (MFB). In the liver, the major cell types that contribute to MFB formation are hepatic stellate cells (HSCs) and portal fibroblasts (PFs). Residing in the space of Disse, HSCs are the principal cell type responsible for collagen synthesis in response to liver injury or changes in ECM stiffness [60]. HSCs are also activated by various mediators released from Kupffer cells, the liver-resident macrophage population, and include transforming growth factor-beta (TGF-*β*) and tumor necrosis factor-alpha (TNF-*α*) [61]. In addition, produced by multiple cells types in liver, connective tissue growth factor (CTGF) promotes the activation of HSCs [62]. When HSCs are activated, they convert from quiescent cells into proliferative, fibrogenic, and contractile MFB and release a variety of inflammatory chemoattractants such as monocyte chemoattractant protein-1 (MCP-1) to recruit monocytes to the liver [63]. PFs are found in the portal tract area and play a predominant role in biliary fibrosis [64]. Although both cell types express alpha smooth muscle actin (*α*SMA) upon activation, research suggests that the MFB population that contributes to CHF/ARPKD is likely derived from PFs [65]. Similar to HSCs, TGF-*β* and CTGF are involved in the activation of PFs [66–68]. By contrast, TNF-*α* does not seem important for PF activation or fibrogenic potential [64]. In addition to their role in biliary fibrosis, activated portal MFB regulate cholangiocyte proliferation through activating P2Y receptors on bile duct epithelia [69]. Following the activation of HSCs or PFs to MFB, two major events occur which promote fibrogenesis. First, activated MFB directly increase the synthesis and deposition of ECM proteins. Second, the MFB proliferate and amplify the fibrotic response [70].

Matrix degradation is an important mechanism to reverse fibrosis or cirrhosis and can restore normal liver architecture. Two kinds of matrix degradation mechanisms exist: “pathologic matrix degradation” that disrupts low density matrix and “restorative matrix degradation” that degrades excess scar [71]. Matrix remodeling is carried out through a fine balance between activities of MMPs and their inhibitors. MMPs are a family of enzymes secreted as proenzymes and are activated by proteolytic cleavage. They play a pivotal role in the regression of liver fibrosis by degrading ECM and inducing MFB apoptosis. Expression of tissue inhibitors of metalloproteinase 1 (TIMP-1) promotes fibrosis, first, by inhibiting MMP activity, and, second, by inhibiting MFB apoptosis [72].

The connection between cyst growth and fibrosis in CHF/ARPKD is thus far unclear. Cystogenesis may be the initial event that disrupts normal ECM remodeling and induces fibrogenesis. This hypothesis is supported by the fact that HSCs and PFs are activated and differentiate into MFB when microenvironmental stiffness increases [66, 73]; enlarging cysts may promote this increase in mechanical stiffness and facilitate PF and HSC activation. Recent evidence suggests that bile duct epithelia directly regulate PF proliferation and PF transdifferentiation to MFB via release of MCP-1 [74]. These data indicate that release of MCP-1 is likely an additional link between cyst growth and fibrosis.

Compared to what has been established regarding development of hepatic fibrosis, little is known about renal fibrosis regarding the mechanisms and consequences in ARPKD. Although the patients exhibit very different renal symptoms, they always develop some degree of interstitial fibrosis accompanied with renal cysts [75]. In PCK rats, the renal interstitial fibrosis is not evident until 70 days of age, and the renal disease is more severe in males than in females [29].

## 7. Mechanisms of Inflammation in ARPKD

In addition to cyst growth and fibrosis, inflammation is another pathological feature of CHF/ARPKD. Although inflammation in the liver has not been well studied as that in the kidney, immune cells such as mast cells (MC) are found accumulated in the pericystic areas in livers of human CHF/ARPKD patients [76]. Consistently, we have observed pericystic MC infiltration in PCK rats (data not shown). Inhibition of MC degranulation and histamine release with cromolyn sodium, a MC stabilizer, decreases cholangiocyte proliferation in bile duct ligation-induced cholestasis [77], suggesting MC may also contribute to cholangiocyte-derived, CWEC proliferation. Moreover, upregulation of genes involved in innate immune responses, including activated complement protein 3 (C3a) and the MCP-1 receptor, is detected in* cpk* mice [78]. Taken together, these data suggest that activation of innate immune effector cells and associated proteins contributes to progression of PKD in general as well as in CHF/ARPKD.

Although PKD is not primarily considered an inflammatory disorder, accumulating evidence suggests that inflammation occurs in the early stage of the disease and may also drive disease progression. For example, macrophage infiltration is found in the renal interstitium in human ADPKD patients with kidney failure [79]. Activated macrophages stimulate vascular endothelial cell proliferation* in vitro* [80]. Further, TNF-*α*, an inflammatory cytokine, is present in renal cyst fluid of human ADPKD and induces renal cyst formation through regulating polycystin-2 [81]. In ARPKD, M2-like macrophages are present in kidneys of patients and in* cpk* mice [82], supporting a role of macrophages in the progression of ARPKD. Work from others has found increased MCP-1 in kidneys [83] from PCK rats. Consistent with a role for MCP-1 and macrophage infiltration in progression in PKD, inhibition of MCP-1 synthesis with bindarit reduces renal inflammation and renal dysfunction but did not attenuate cyst growth [83]. Whether or not MCP-1 depletion affected hepatic inflammation, cyst growth, or fibrosis was not evaluated in this study.

**Figure 1:**
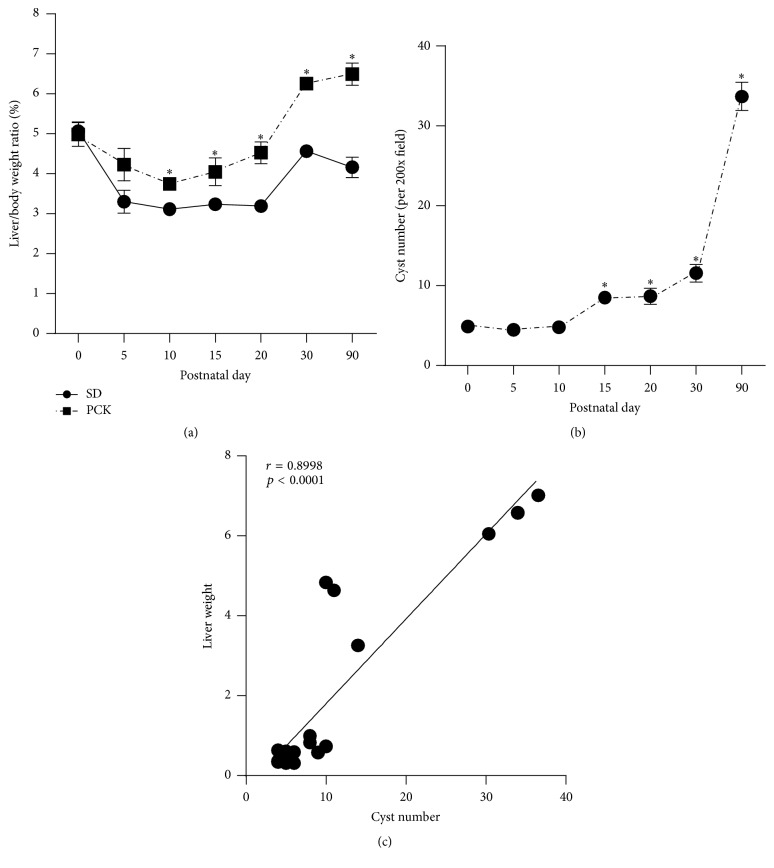
Characterization of hepatic cyst growth in PCK rats. (a) Liver/body weight ratio (as a percent of body weight) in Sprague-Dawley (SD) and polycystic kidney (PCK) rats was calculated from postnatal day (PND) 0 to PND 90. *∗*, significantly different than SD rats at the indicated time point (*p* < 0.05). (b) Cyst number was quantified in 200x, hematoxylin and eosin-stained images from PCK rats between PND 0 and PND 90. *∗*, significantly different than PND 0 (*p* < 0.05). (c) Pearson correlation of the relationship between liver weight and cyst number in PCK rats from PND 0 to PND 90. In all cases, *n* = 2–4 rats per genotype per time point.

**Figure 2:**
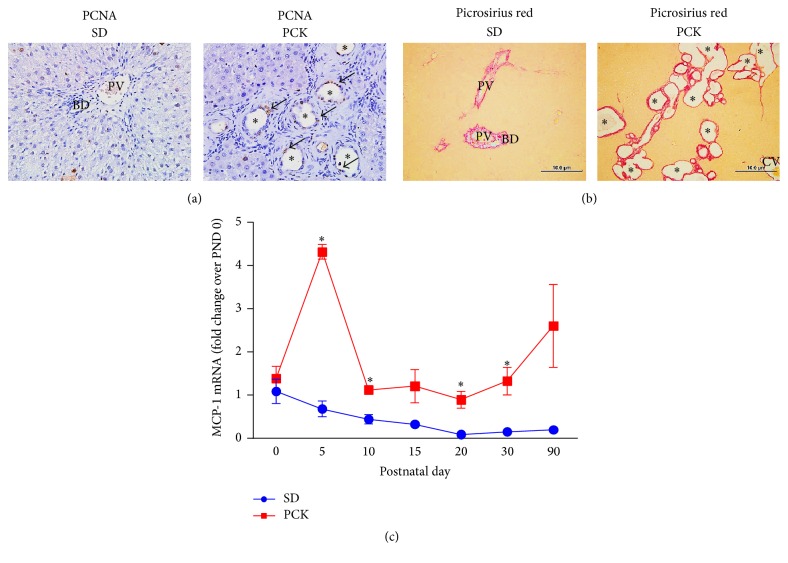
Cyst wall epithelial cell proliferation, fibrosis, and inflammation in PCK rats. (a) Hyperproliferation of cystic epithelia in polycystic kidney (PCK) rats. Hepatic PCNA content was assessed in Sprague-Dawley (SD) rats and PCK rats by immunohistochemistry. Images were taken at 400x magnification. Arrows indicate PCNA-positive cyst wall epithelial cells (CWECs) and asterisks indicate hepatic cysts. (b) Pericystic fibrosis in PCK rats. Extracellular matrix was localized in livers from SD and PCK rats by picrosirius red staining. Images were taken at 100x magnification. A scale bar (100 *μ*M) is included in each image. PV = portal vein, CV = central vein, and asterisks = cysts (some, but not all are indicated). (c) Inflammation in PCK rats. Monocyte chemoattractant protein-1 (MCP-1) transcripts in SD (blue circles) and PCK (red squares) rats from postnatal days (PND) 0 to 90 were measured in liver using real-time PCR as a surrogate marker for inflammation. The data are expressed as fold change over control (SD) at PND 0. All images are representative of *n* = 2–4 rats at each time point and data are graphed as means plus standard error of the mean. ^*∗*^ *p* < 0.05 between SD and PCK rats at the time points indicated.

We characterized hepatic cyst development in PCK rats by calculating liver/body weight ratio (%) and quantifying cyst number, compared to SD rats from postnatal days (PND) 0 to 90 (Figure 1). Consistent with the work of others [29], our data suggest that PCK rats had increased liver/body weight ratios (Figure 1(a)) which was paralleled by an increase in the number of cysts from PND 10 onward (Figure 1(b)). Consistently, a positive correlation exists between liver weight and cyst number, further supporting a relationship between these disease-related parameters (Figure 1(c)). To characterize CWEC proliferation, we performed immunohistochemical staining for proliferating cell nuclear antigen (PCNA), a nuclear protein expressed in G1-M phase [84], in SD and PCK rat liver. In PCK rat liver, PCNA was strongly expressed in CWECs compared to limited PCNA-positive staining in cholangiocytes in SD rat liver (Figure 2(a)). Additional studies suggest that pericystic fibrosis occurs in parallel with biliary dysgenesis in PCK rats [29, 39]. By using picrosirius red staining to localize ECM, we found an increased level of fibrillar collagen deposition in PCK rat liver compared to that observed in SD rats (Figure 2(b)). In addition to cyst growth and pericystic fibrosis in PCK rat liver, we recently measured hepatic MCP-1 transcript levels in SD and PCK rats as a surrogate marker of hepatic inflammation and driver of fibrosis. The expression of MCP-1 was elevated in PCK rat liver compared to SD rats at PND 5, 10, 20, and 30, which is consistent with the work showing that MCP-1 is upregulated in PCK rat kidney [83]. While further studies are required, these data suggest that increased inflammation, perhaps mediated by macrophages or PFs, is a potent contributor to CHF/ARPKD progression.

## 8. “Pathogenic Triumvirate” in CHF/ARPKD: Insights into the Development of New Therapies

**Figure 3:**
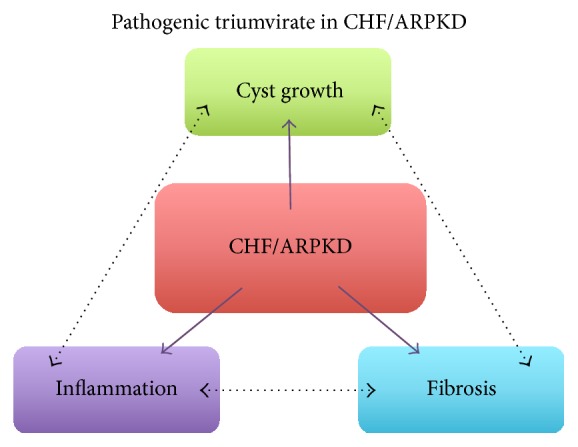
A “pathogenic triumvirate” in congenital hepatic fibrosis (CHF) in autosomal recessive polycystic kidney disease (ARPKD). Published data suggest that cyst growth, fibrosis, and inflammation drive CHF/ARPKD (solid purple arrows). We propose that relationships exist between cyst growth, fibrosis, and inflammation which drive progression of CHF/ARPKD (black, double-headed arrows with broken lines). Targeting a single pathway which drives each of the triumvirate members, or targeting multiple members concurrently, may provide better therapeutic strategies than if targeting any one member in isolation.

Although mutations in the human* PKHD1* gene, or mutations in* PKHD1* orthologs in rats and mice, are required for development of CHF/ARPKD, other factors are also involved in disease progression. It is from a review of the published literature summarized in this paper that we propose a “pathogenic triumvirate” in CHF/ARPKD which includes three factors, cyst growth, fibrosis, and inflammation, as mediators which contribute to disease progression (Figure 3). Despite recent advances in our understanding of what contributes to the pathology of CHF/ARPKD, less is known about the molecular mechanisms regulating cyst growth, progression of fibrosis, and how inflammation contributes to these interrelated processes. Furthermore, whether or not a common mechanism drives members of the “pathogenic triumvirate” is also not known. We propose that leveraging what we do know about the CHF/ARPKD pathogenesis in the context of the pathogenic triumvirate will lead the way to new research and, possibly, new therapies for this disease. For example, finding a central mechanism that regulates all three components would be an attractive target for the development of new pharmacologic approaches to manage CHF/ARPKD. Alternatively, if a common mechanism does not exist, therapeutically targeting each member of the triumvirate concurrently may be a favorable approach.

## 9. Summary and Conclusions

CHF/ARPKD is a genetic disease, but many factors contribute to its pathology and progression. Cyst growth is mainly regulated by cAMP and intracellular [Ca^2+^] through stimulating cholangiocyte proliferation and fluid secretion. While these signals are clearly important in CHF/ARPKD, additional molecules and pathways which drive cell proliferation in cystic disease are being discovered and require further exploration. Development of hepatic fibrosis depends on the balance between ECM synthesis and degradation. Much research is needed not only to understand the role of the ECM in disease progression but also to define which cells are responsible for development of fibrosis in the first place (e.g., HSCs, PFs). Even less is known regarding the role inflammation plays in CHF/ARPKD. Future research should focus on identifying immune cell types and inflammatory mediators found in CHF/ARPKD and elucidating their roles in protection or promotion of disease. To date, the treatments for ARPKD are very limited and rely mostly on liver/kidney transplantation. Our “pathogenic triumvirate” identifies three target areas, cyst growth, fibrosis, and inflammation, which influence CHF/ARPKD progression. We believe that an integrated approach targeting each member of the pathogenic triumvirate at the same time, either by way of a common core pathway or three independent pathways, is required to improve therapeutic strategies for CHF/ARPKD. Further studies, utilizing the PCK rat or relevant mouse models of CHF/ARPKD, should strive to implement this idea in the preclinical arena.

## Figures and Tables

**Table 1:** Current rodent models of ARPKD.

Model	Species	Liver phenotype	Kidney phenotype	Other phenotypes	Reference
PCK	Rat	Cysts and fibrosis	Cysts	Pancreatic cysts	[29]
BALB/c-*cpk/cpk*	Mouse	Cysts and fibrosis	Cysts	Pancreatic cysts and fibrosis	[33]
C57BL/6J-*cpk/cpk*	Mouse	No liver disease	Cysts	None	[34]
Pkhd1^del2/del2^	Mouse	Cysts and fibrosis	Cysts in female	Pancreatic cysts	[35]
Pkhd1^LSL(−)/LSL(−)^	Mouse	Cysts and fibrosis	Cysts in female	Unknown	[36]
Pkhd1exon40	Mouse	Cysts and fibrosis	No kidney disease	Portal hypertension	[37]
Pkhd1^lacZ/lacZ^	Mouse	Cysts and fibrosis	Cysts	Pancreatic and gall bladder cysts	[5]
Pkhd1^del4/del4^	Mouse	Cysts and fibrosis	No kidney disease	Pancreatic cysts, splenomegaly	[38]
